# Na^+^/H^+^ Exchanger Isoform 1-Induced Osteopontin Expression Facilitates Cardiomyocyte Hypertrophy

**DOI:** 10.1371/journal.pone.0123318

**Published:** 2015-04-17

**Authors:** Iman A. Mohamed, Alain-Pierre Gadeau, Larry Fliegel, Gary Lopaschuk, Mohamed Mlih, Nabeel Abdulrahman, Natasha Fillmore, Fatima Mraiche

**Affiliations:** 1 College of Pharmacy, Qatar University, Doha, Qatar; 2 University of Bordeaux, Adaptation Cardiovasculaire à L'ischémie, UMR1034, Pessac, France; 3 Department of Biochemistry, Faculty of Medicine and Dentistry, University of Alberta, Edmonton, Alberta, Canada; 4 Mazankowski Alberta Heart Institute, University of Alberta, Edmonton, Alberta, Canada; University of Western Ontario, CANADA

## Abstract

Enhanced expression and activity of the Na^+^/H^+^ exchanger isoform 1 (NHE1) has been implicated in cardiomyocyte hypertrophy in various experimental models. The upregulation of NHE1 was correlated with an increase in osteopontin (OPN) expression in models of cardiac hypertrophy (CH), and the mechanism for this remains to be delineated. To determine whether the expression of active NHE1-induces OPN and contributes to the hypertrophic response *in vitro*, cardiomyocytes were infected with the active form of the NHE1 adenovirus or transfected with OPN silencing RNA (siRNA-OPN) and characterized for cardiomyocyte hypertrophy. Expression of NHE1 in cardiomyocytes resulted in a significant increase in cardiomyocyte hypertrophy markers: cell surface area, protein content, ANP mRNA and expression of phosphorylated-GATA4. NHE1 activity was also significantly increased in cardiomyocytes expressing active NHE1. Interestingly, transfection of cardiomyocytes with siRNA-OPN significantly abolished the NHE1-induced cardiomyocyte hypertrophy. siRNA-OPN also significantly reduced the activity of NHE1 in cardiomyocytes expressing NHE1 (68.5±0.24%; *P<0*.*05*), confirming the role of OPN in the NHE1-induced hypertrophic response. The hypertrophic response facilitated by NHE1-induced OPN occurred independent of the extracellular-signal-regulated kinases and Akt, but required p90-ribosomal S6 kinase (RSK). The ability of OPN to facilitate the NHE1-induced hypertrophic response identifies OPN as a potential therapeutic target to reverse the hypertrophic effect induced by the expression of active NHE1.

## Introduction

Cardiovascular diseases (CVDs) are one of the leading causes of death worldwide despite the advances in treatment [1]. Cardiac hypertrophy (CH) is a condition characterized by the enlargement of cardiomyocytes following chronic and acute morbidities [2]. CH that is left unresolved results in left ventricular dysfunction and heart failure [3]. Previous reports have suggested that increased activity of the Na^+^/H^+^ exchanger (NHE) isoform 1, a cardiac specific isoform of the NHE exchanger family, is involved in CH [4,5,6] and ischemia/reperfusion (I/R) injury [7]. NHE1 is a ubiquitously expressed housekeeping glycoprotein that maintains intracellular pH through exchange of one intracellular H^+^ for one extracellular Na^+^ [8,9]. The implication of NHE1 in CH has been highlighted in guanylyl cyclase-A knockout (GC-A KO) mice, which demonstrated CH and enhanced NHE1 activity [10]. In addition, complimentary genetic evidence for the key role of NHE1 in CH has been demonstrated in transgenic mice expressing a cardiac specific active form of NHE1, rather than a wild type form of NHE1, had an exacerbated hypertrophic response [11]. Furthermore, pharmacological inhibition of NHE1 activity was sufficient to reduce isoproterenol-induced CH [12,13]. The encouraging evidence has led to clinical trials in which an NHE1 inhibitor was used in patients with myocardial infarctions. These studies demonstrated severe cerebrovascular side effects leading to enhanced mortality [14]. Despite the complications associated with the administration of NHE1 inhibitors, delineating the molecular mechanisms downstream of NHE1 activation in the setting of CH is necessary to develop specific strategies to indirectly inhibit NHE1.

Recent studies involving transgenic mice expressing active NHE1 [5] and mineralocorticoid/salt-induced cardiac fibrosis in rats [15] were associated with the activation of NHE1 and osteopontin (OPN) protein and mRNA expression. Moreover, Sgk1-induced CH and upregulation of NHE1 were correlated with an increase in OPN expression [6,16]. OPN, a matricellular protein involved in mediating inflammation and contributing to the pathogenesis of CH [17–19], is increased under conditions of hypoxia [20] and following stimulation with endothelin, norepinephrine, angiotensin II and aldosterone [21,22], conditions in which NHE1 also appears to be active. In addition, inhibition of NHE1 was correlated with a significant reduction in OPN expression [6,15]. These findings suggest a molecular link between enhanced NHE1 activity and OPN, a pathway that has never been clearly defined.

OPN has been suggested to function through the calcineurin/nuclear factor activated T cells (CaN/NFAT) pathway [23,24] and GATA4 [17]. The CaN/NFAT pathway has also been implicated in NHE1 induced CH [25,26]. Osteopontin [17,27] and NHE1 [28–30] have also been shown to activate the mitogen activated protein kinases (MAPK) and phosphatidylinositide-3-kinases (PI3K) in a cell-specific manner. Therefore, OPN appears to be a key regulator of similar hypertrophic signaling pathways activated during NHE1-induced cardiomyocyte hypertrophy.

Our study was undertaken to delineate the cellular mechanism by which NHE1 induces OPN and its contribution to the NHE1-mediated hypertrophic response. We demonstrated for the first time that increased NHE1 activity stimulated OPN, which induced an amplification loop promoting CH associated with altered NHE1 activity.

## Materials and Methods

Materials. All routine chemicals were purchased from BD Biosciences (San Jose, CA), Fisher Scientific (Ottawa, ON) or Sigma (St. Louis, MO). EMD87580 was a generous gift of Dr. N. Beier of Merck KGaA (Frankfurt, Germany). RSK inhibitor (BI-D1870) was obtained from the University of Dundee (Scotland, United Kingdom). Primary antibodies used for western blotting including mouse monoclonal anti-HA-tag (#2376, 6E2), rabbit polyclonal anti-extracellular signal regulated kinase (ERK)1/2 (#9102), mouse monoclonal phospho-ERK1/2 (Thr^202^/Tyr^204^) (#9106), rabbit polyclonal protein kinase B (Akt) (#9272), rabbit polyclonal phospho-Akt (Ser^473^) (#9271) and phospho-p90RSK (Ser^380^) (#9341) were purchased from Cell Signaling Technology (Pickering, ON); mouse monoclonal anti-NHE1 was from BD Biosciences Pharmingen (San Diego, CA), rabbit polyclonal anti-RSK 1 (C-21) (sc-231) and RSK 2 (C-19) (sc-1430), goat polyclonal GATA4 (C-20) (sc-1237) and rabbit polyclonal phospho-GATA4 (Ser^262^) (sc-32823) were from Santa Cruz Biotechnology (Santa Cruz, CA); rabbit polyclonal anti-OPN (ab8448), α-tubulin (ab4074) and glyceraldehyde-3-phosphate dehydrogenase GAPDH (ab9485) antibodies were from Abcam (Cambridge, MA). Secondary polyclonal antibodies including goat-anti-mouse, goat-anti-rabbit and donkey-anti-goat, conjugated to horseradish peroxidase were purchased from Jackson ImmunoResearch (West Grove, PA) or Abcam.

Adenoviral preparation. The pAdTrack plasmid was used to engineer the OPN and NHE1 containing adenoviruses as previously described [9,11]. Both the human NHE1 (PYN4^+^) (a generous gift from Dr. Larry Fliegel, University of Alberta, Edmonton, Alberta) [11] and mouse OPN (BC057858) (a generous gift from Dr. Alain Gadeau, INSERM, Pessac, France) [18] plasmids contained the hemagglutinin (HA) tag and green fluorescent protein (GFP). The NHE1 plasmid also contained mutations in the Lys^641^, Arg^643^, Arg^645^ and Arg^647^ sites (to glutamic acid) that render the protein constitutively active [11].

Isolation and culture of neonatal rat ventricular cardiomyocytes. All experimental procedures involving neonatal rats were in accordance with guidelines set out by the Canadian Council on Animal Care and carried out in Edmonton, Alberta, Canada and the Institutional Animal Care and Use Committee at Qatar University, Doha, Qatar. The Institutional Animal Care and Use Committee at Qatar University, Doha, Qatar has specifically approved this project (Research Ethics Approval Number: QU-IACUC 007/2012). Ethical approval was received from the University of Alberta, Edmonton, Alberta, Canada. Cardiomyocytes from 1–3 day-old neonatal rat pups were isolated as described previously [31]. Briefly, hearts were extracted and the isolated ventricles were digested using (2%) DNase (w/v), (0.5%) collagenase (w/v), and (2%) trypsin (w/v). The digested tissue was centrifuged at 800 rpm for 1 minute at 37°C in isolating media (DMEM/F12 1:1 media, 20% fetal bovine serum (FBS), 1% penicillin/streptomycin, and 50 μg/mL gentamycin mixed stock solution). The pellet was resuspended in plating media (DMEM/F12, 11% horse serum, 5% FBS, 1% penicillin/streptomycin, and 50 μg/mL gentamycin mixed stock solution) and incubated at 37°C for 2 hours. The cell suspension was centrifuged at 1,000 rpm for 2 minutes at 37°C and the remaining pellet was resuspended in plating media. Cardiomyocytes were plated on Primaria-coated dishes (Falcon) at a density of 2.0 × 10^6^ cells/well. NRVMs were cultured for 48 hours at 37°C in a humidified atmosphere (95% O_2_-5% CO_2_) prior to infection with the respective adenoviruses.

Differentiation and culturing of H9c2 cardiomyocytes. H9c2 myoblasts are a clonal cell line derived from the embryonic BD1X rat heart tissue and were obtained from European Collections of Cell Cultures. They have been reported to display comparable hypertrophy-associated traits to primary cultures of cardiomyocytes when stimulated with hypertrophic agents [32]. H9c2 myoblasts were initially cultured in DMEM/F12 1:1 culture media supplemented with 10% FBS and 1% penicillin/streptomycin at 37°C in a humidified atmosphere (95% O_2_-5% CO_2_) [33]. H9c2 myoblasts were differentiated into the cardiac phenotype by culturing in DMEM/F12 1:1 supplemented with 1% horse serum, 1% penicillin/streptomycin and 0.1μM all-trans-retinoic acid over a five day period [34]. Following differentiation, H9c2 cardiomyocytes were cultured in DMEM/F12 1:1 culture media supplemented with 1% FBS and devoid of antibiotics for 24 hours prior to infection with the adenoviruses and/or transfection with siRNA OPN.

Adenoviral infection or siRNA transfection of cardiomyocytes. Cardiomyocytes were infected for 24 hours with active NHE1 expressing adenovirus, the OPN adenovirus or an adenovirus containing GFP using a multiplicity of infection (MOI) of 10, 20 or 30 as indicated. Lipofectamine 2000 (Invitrogen) was used to transfect cardiomyocytes with 100 nM siRNA OPN (siRNA OPN-1 5’-GAUGAUAGGUAUCUGAAAUTT-3’ and siRNA OPN-2: 5’-CGGAUGACUUUAAGCAAGATT-3’) or 30 nM universal scrambled siRNA (Eurogentec, http://www.eurogentec.com) for 24 hours according to the manufacturer’s instructions. Cardiomyocytes co-transfected with the active form of the NHE1 adenovirus and siRNA were transfected with siRNA-OPN 4 hours post infection with the active form of the NHE1 adenovirus. Cardiomyocytes infected/transfected with the respective adenovirus in the presence and absence of siRNA were maintained at 37°C in a humidified atmosphere (95% O_2_-5% CO_2_) for 24 hours prior to cell lysis and other assays.

Phenylephrine and BI-D1870 treatment of cardiomyocytes. H9c2 cells were treated with either vehicle or 100 μM phenylephrine (PE) for 30 minutes in the presence and absence of 10 μM BI-D1870. Treated cardiomyocytes were maintained at 37°C in a humidified atmosphere (95% O_2_-5% CO_2_) for 24 hours prior to cell lysis.

Western blot analysis. Cardiomyocytes were lysed 24 hours post-infection and/or siRNA OPN transfection using radio-immunoprecipitation protein assay (RIPA) buffer as described earlier [11]. Cell lysates were centrifuged at 14,000 rpm at 4°C and the supernatant containing the proteins were collected. The total amount of protein present in each sample was quantified using the DC protein assay kit according to the manufacturer’s instructions. For determination of protein expression by western blotting, up to 40 μg of protein was resolved on 10% SDS-PAGE and transferred on to nitrocellulose membranes. NHE1 and OPN protein expression were normalized to GAPDH or α-tubulin. Phosphorylated GATA4 protein expression was normalized to total protein expression. For the p-ERK/ERK, p-RSK/RSK and p-Akt/Akt the phosphorylated kinase expression were normalized to the respective kinase total protein expression. Cells were sonicated in 1 mL of MAPK cell lysis buffer (mM (50 Na-pyrophosphate, 50 NaF, 50 NaCl, 5 EDTA, EGTA, 0.1 sodium orthovandate, 10 Hepes pH 7.4, 0.5 PMSF), 0.1% Triton X-100, 10 mg/mL leupeptin) [35]. All primary antibodies were incubated in a dilution of 1:1000–2000, while secondary antibodies were diluted at 1:5000. Immunoreactive proteins were visualized using enhanced chemiluminescence (Amersham Biosciences) and imaged and quantified using the Alpha Innotech FluorChem Imager (R&D Systems).

Measurement of NHE1 activity. Cardiomyocytes plated on coverslips were loaded with 3 μg/mL pH sensitive dye 2,7-bis(carboxyethyl)-5(6)-carboxyfluorescein acetoxymethyl ester (BCECF-AM). The change in H^+^ concentration was measured using a PTI Deltascan spectrofluorometer (Photon Technology International; London, Ontario). The excitation wavelengths were set at 502.5 nm and 440 nm and the emission wavelength was set at 528.7 nm [36]. The coverslip was initially maintained in a pre-warmed solution of Na^+^-normal buffer (mM (135 NaCl, 5 KCl, 1.8 CaCl_2_, 1 MgSO_4_, 5.5 Glucose, 10 HEPES) at 37°C and was then pulsed with 50 mM ammonium chloride to induce an acid load [36]. Following acidification, the coverslip was placed in Na^+^-normal buffer to allow the cardiomyocytes to recover. Each coverslip was equilibrated in a three-step pH calibration buffer solution containing 135 mM N-methyl-glucamine and KCl, 10 μM nigericin and adjusted to a pH of 8, 7 or 6. The three-step pH calibration was used to generate a standard curve in which the fluorescence output measurements were converted into intracellular pH [16]. The initial rate of recovery following an induced acid load was measured and used as an indicator of the NHE1 activity.

Measurement of cell surface area. The average surface area of 50–70 randomly selected infected/transfected cardiomyocytes was measured out of 3–4 experiments. The cardiomyocytes were visualized with an inverted microscope equipped with a monochrome digitalized camera using 20X magnification. The surface area was determined using the AxioVision Imaging Software (Carl Zeiss Microimaging, New York, NY).

Measurement of protein content. Protein content was measured as described previously [37]. Briefly, infected/transfected cardiomyocytes were washed twice in 1 x PBS and collected by trypsinization. The total number of cardiomyocytes was calculate using a Countess® Cell Counting Chamber Slide (Invitrogen). Protein concentration of infected/transfected cardiomyocytes lysed in RIPA buffer was measured using the DC protein assay kit (Biorad). Protein content was determined by dividing the total amount of protein (μg) by the total number of cardiomyocytes.

Expression of ANP and OPN mRNA using Reverse Transcription-Polymerase Chain Reaction. RNA was extracted from cardiomyocytes using the Total RNA Purification Kit (Norgen). Total RNA (1 μg) was reverse transcribed into cDNA using SuperScript® III First Strand Synthesis SuperMix (Invitrogen). 100 ng of cDNA was amplified using sense 5′-CTGCTAGACCACCTGGAGGA-3′, antisense 5′-AAGCTGTTGCAGCCTAGTCC-3′ and sense 5’-CAGTCGATGTCCCTGACGG-3’, antisense 5’-GTTGCTGTCCTGATCAGAGG-3’ ANP and OPN primer sequences, respectively, using a 2x PCR Master Mix (Norgen) [38,39]. β-actin cDNA was primed with sense 5′-ACGCAGCTCAGTAACAGTCC-3′ and antisense 5′-AGATCAAGATCATTGCTCCTCCT-3′ primer sequence and used to normalize mRNA expression. Following an initial denaturation of 3 minutes at 95°C, the samples were denatured at 95°C for 30 seconds, annealed at 60°C for 30 seconds and extended at 72°C for 1 minute for 35 cycles. A final extension of 72°C for 5 minutes was performed in order to ensure the maximum recovery of products. ANP, OPN and β-actin were quantified using the Alpha Innotech FluorChem Imager. The changes in ANP and OPN mRNA levels were normalized to β-actin and then to control.

Statistics. All values expressed were compared to control or NHE1 infected cardiomyocytes ± SEM (%). Student’s *t* test was used to compute differences between groups where a *P*<0.05 was considered a significant difference.

## Results

### Activation of NHE1 stimulates OPN expression

**Fig 1:**
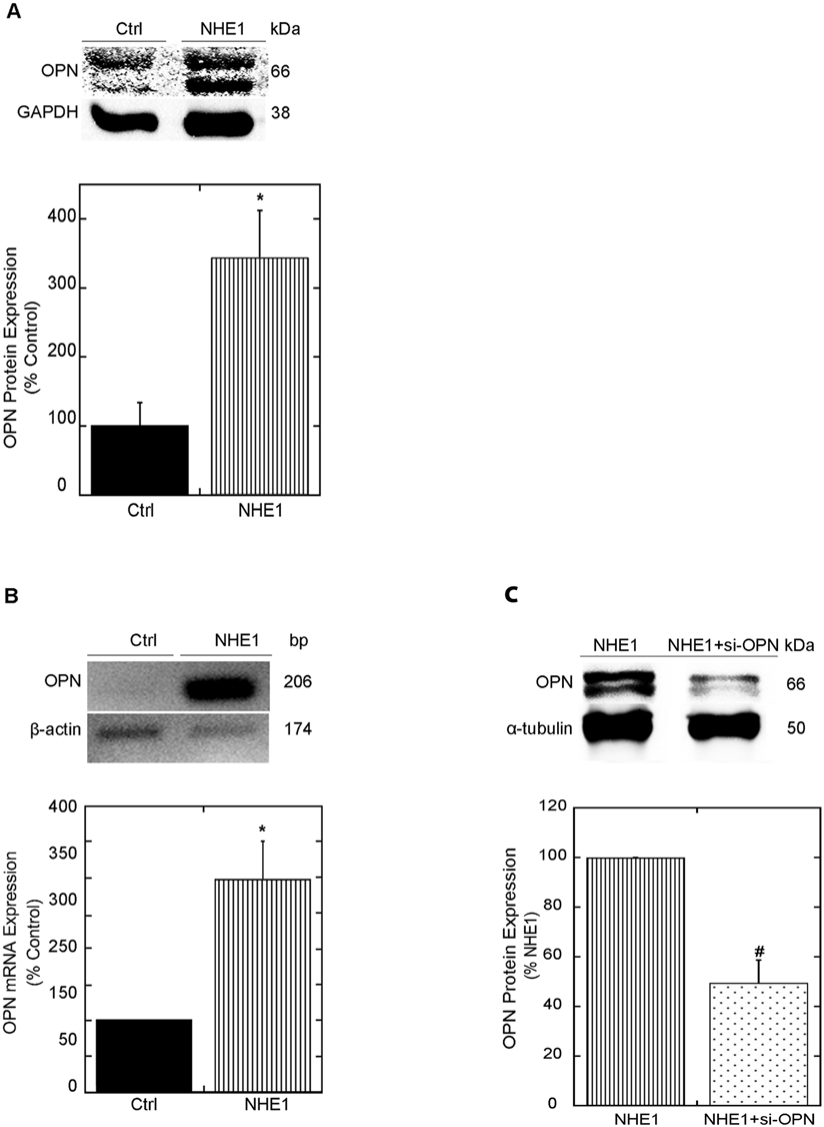
Upregulation of NHE1 in cardiomyocytes enhances OPN expression. A: Upper panel, representative western blot of OPN protein expression in cell lysates of NRVMs infected with GFP (control) or active NHE1 adenovirus for 24 hours. Immunoblotting was against OPN (doublet at 66 kDa) and GAPDH (38 kDa). Lower level, quantification of relative levels of *total* OPN protein expression (n = 4; representative of 2–3 preparations). B: Upper panel, representative DNA gel of OPN mRNA expression in H9c2 cardiomyocytes infected with GFP (control) or active NHE1 adenovirus for 24 hours using an MOI of 20 and 30, respectively. cDNA amplification was against OPN (206 bp) and β-actin (174 bp). Lower level, quantification of OPN mRNA expression in H9c2 cardiomyocytes normalized to β-actin (n = 6–7; representative of 4 experiments). Results expressed as % of control (GFP) ± %SEM. *p < 0.*05* vs. control. C: Upper panel, representative western blot of OPN protein expression of H9c2 cardiomyocytes infected with NHE1 adenovirus (30 MOI) in the presence and absence of 100nM siRNA OPN for 24 hours. Immunoblotting was against OPN (doublet at 66 kDa) and α-tubulin (50 kDa); lower panel, quantification of relative levels of OPN protein expression (n = 6–9; representative of 2–3 experiments). Results are expressed as % of NHE1 ± %SEM. *p < 0.*05* vs. NHE1 + siRNA.

Previous reports have demonstrated a simultaneous upregulation of NHE1 and OPN in models of CH [5,6,15,16]. In order to determine whether NHE1 activation induces OPN expression during cardiomyocyte hypertrophy, H9c2 cardiomyocytes and NRVMs were infected with adenoviral vectors coding a constitutively active NHE1 for 24 hours. An enhanced green fluorescent protein (GFP)-expressing adenovirus under the control of the same promoter of the NHE1 and OPN adenoviruses served as a control. Our results indicated that infection of H9c2 cardiomyocytes or NRVMs with the NHE1 adenovirus successfully overexpressed the HA-tagged protein (S1A Fig and S2A Fig), as well as total NHE1 protein expression (S1B Fig and S2B Fig), an effect that was not observed in cardiomyocytes infected with the GFP adenovirus alone. OPN protein and mRNA expression were then examined in cardiomyocytes infected with the active form of the NHE1 adenovirsu. OPN protein expression was examined in NRVMs 24 hours post infection. Our results revealed that expression of the OPN protein, appearing as a doublet at 66 kDa [17,40], was significantly elevated in cardiomyocytes expressing the active form of the NHE1 adenovirus (342.7%±69.22% vs. 100.0±33.93% control; *P*<0.05 (Fig 1A). OPN mRNA expression was significantly increased in cardiomyocytes expressing active NHE1 in H9c2 cardiomyocytes (296.3%±53.60% vs. 100.0% control; *P*<0.05) (Fig 1B). To identify the role of OPN in the NHE1 mediated hypertrophic effect we induced NHE1 activity in cardiomyocytes and blocked the OPN using siRNA (S3B Fig). The remaining portion of our study in which OPN was blocked using siRNA was carried out in H9c2 cardiomyocytes. The H9c2 cell model has been suggested to be a more suitable host for siRNA transfection as it represents a form of immortal mammalian cell line as opposed to NRVMs, which may require electroporation to allow for the uptake of siRNA [41]. Inhibition of OPN with siRNA directed against OPN reduced the NHE1-induced upregulation of OPN protein expression by more than 50% in H9c2 cardiomyocytes at the 24 hour time point (49.5±9.18% vs. 100.0% NHE1; *P*< 0.05) (Fig 1C and S3A Fig). Our results demonstrated for the first time that the upregulation of active NHE1 in cardiomyocytes induced the expression of OPN in both NRVMs and H9c2 cardiomyocytes.

### OPN is critical for NHE1-induced cardiomyocyte hypertrophy

**Fig 2:**
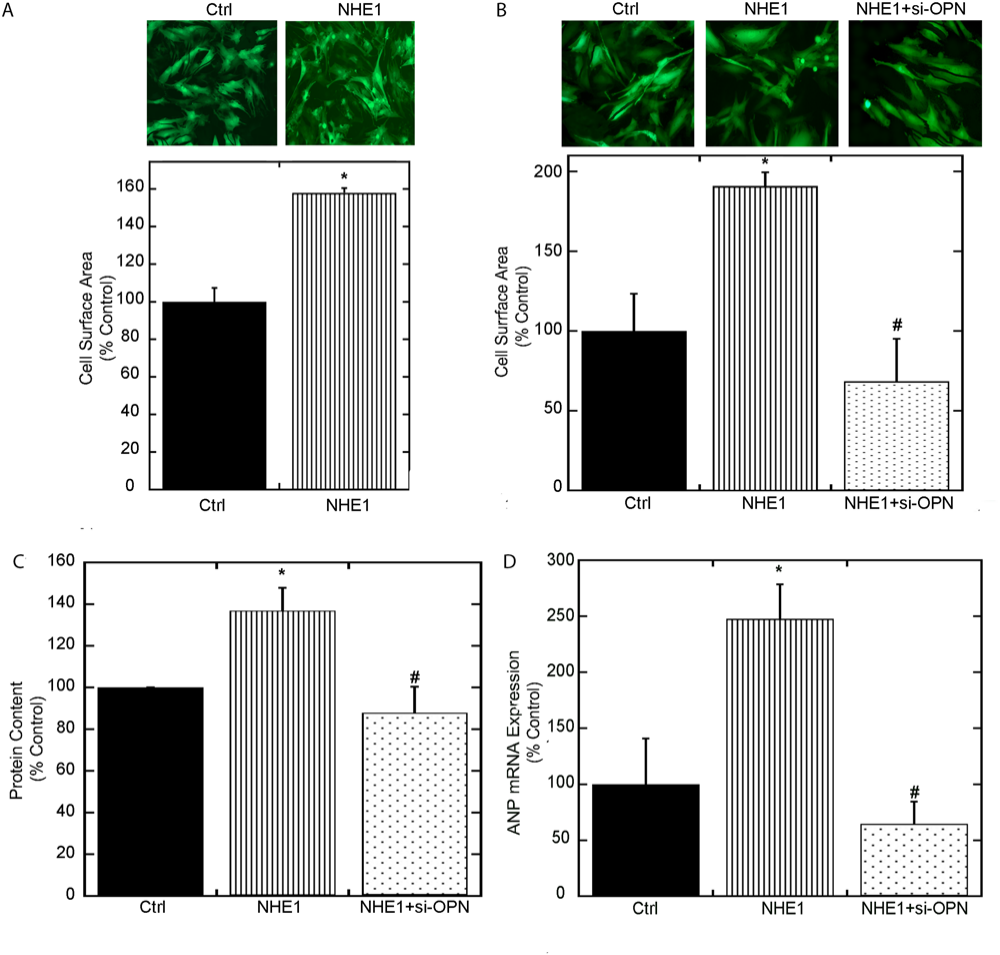
OPN contributes to NHE1-induced cardiomyocyte-hypertrophy. A: upper panel, representative fluorescence microscopy images of NRVMs infected with GFP (control) or active NHE1 adenovirus 24 hours post infection; lower panel, cell surface area of at least 50–70 infected NRVMs from 4–6 individual dishes were measured to represent 3–4 experiments, results expressed as % of control (GFP) ± %SEM. B: Upper panel, representative fluorescence microscopy images of H9c2 cardiomyocytes infected with adenoviruses containing GFP (control) or active NHE1 in the presence and absence of siRNA OPN for 24 hours; lower panel, cell surface area of at least 50–70 H9c2 cardiomyocytes from 4–6 individual dishes were measured to represent 3–4 experiments. C: Protein content of H9c2 cardiomyocytes expressed as μg/10 x 10^6^ cell. D: Quantification of ANP mRNA expression in H9c2 cardiomyocytes normalized to β-actin (n = 6–7; representative of 4 experiments). Results are expressed as % of control (GFP) ± %SEM. *p < 0.05 vs. control, *#* vs. NHE1.

Although OPN has been suggested to mediate CH [17–19], whether OPN contributes to cardiomyocyte hypertrophy induced by elevated expression and activity of NHE1 has not been shown. *In vitro*, cardiomyocytes infected with the OPN adenovirus alone (expressing a three fold increase in OPN mRNA vs. control) did not cause a significant increase in cell surface area (153.2±26.65% of control), protein content (156.5±19.86% of control) or ANP mRNA (141.5±86.65% of control). Transfection of cardiomyocytes with siRNA-OPN alone (in the absence of the NHE1 adenovirus) was also unable to reverse any parameters of cardiomyocyte hypertrophy compared to control (cell surface area (51.9±1170.77% of control), protein content (96.8±17.81% of control) or ANP mRNA (66.3±0.59% of control)). However, cardiomyocytes expressing the active form of NHE1 adenovirus induced cardiomyocyte hypertrophy as indicated by the significant increase in cell area in both the H9c2 cardiomyocytes and NRVMs (Fig 2A and 2B). Total protein content (Fig 2D) and ANP mRNA expression (Fig 2E) were also significantly increased (136.8±11% and 247.7±30.81% of control; *P*<0.05 respectively) in H9c2 cardiomyocytes expressing the active form of the NHE1 adenovirus. The downregulation of OPN by transfection of siRNA directed against OPN in cardiomyocytes expressing active NHE1 fully reversed the NHE1 hypertrophic effect as indicated by the significant reduction in cell surface area (68.5±0.24% vs. 190.9±8.66%; *P*< 0.05), total protein content (87.8±12.58% vs. 136.8±11%, *P*< 0.05) and ANP mRNA expression (64.6±19.9% vs. 247.7±30.81% *P*< 0.05) (Fig 2B–2E). Transfection of H9c2 cardiomyocytes with scrambled siRNA did not alter protein content (121.3±27.48% of control). Our findings reveal for the first time that NHE1-induced OPN expression contributed to the hypertrophic response in cardiomyocytes and that partial inhibition of OPN prevented the NHE1 induced hypertrophic response.

**Fig 3:**
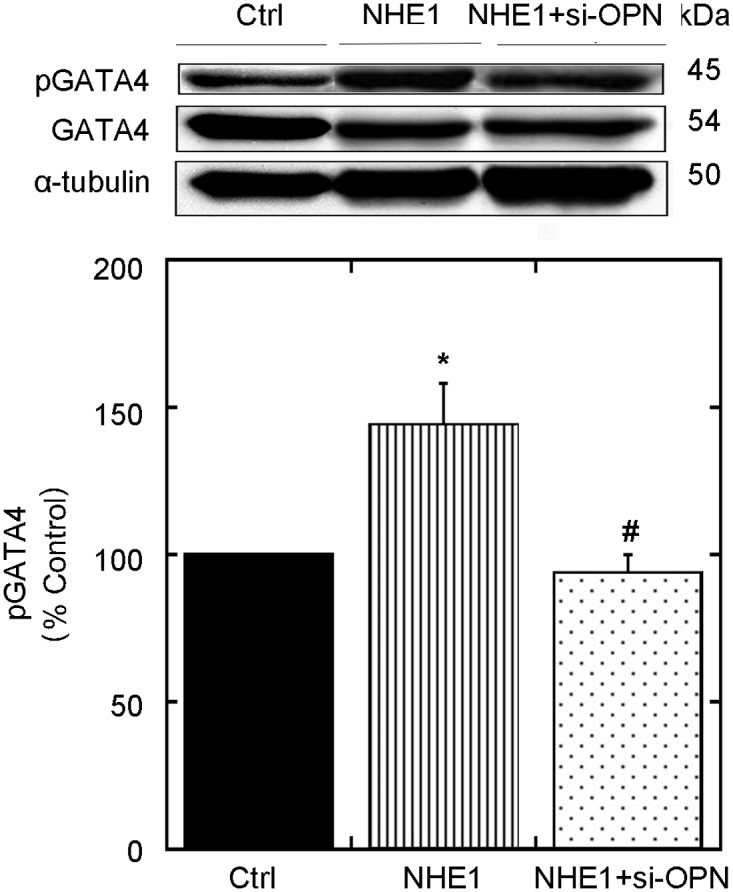
OPN siRNA regresses expression of the GATA-4 hypertrophic transcription factor. Upper panel, representative western blot of relative amounts of phosphorylated and total GATA4 expression in H9c2 cardiomyocytes infected with GFP (control) or active NHE1 in the presence and absence of siRNA OPN. Lower panel: quantification of experiments measuring the ratio of phosphorylated to total GATA4 protein. Results are expressed as % of control (GFP) ± %SEM (n = 4–7; representative of 3–5 experiments). *p < 0.05 vs. control, # vs. NHE1.

The NHE1-mediated hypertrophic effect has previously been demonstrated to be driven at least in part by the CaN/NFAT pathway [25,26]. We tested whether OPN contributes to this pathway by examining the level of GATA4 phosphorylation, a key activator of the CaN/NFAT pathway that leads to the expression of hypertrophic genes. Active NHE1 enhanced phosphorylation of GATA4 (144.0±14.01% of control; *P*<0.05) (Fig 3). GATA4 phosphorylation was significantly reduced following inhibition of OPN by transfection of cardiomyocytes with siRNA compared to cardiomyocytes expressing active NHE1 (93.7±6.31% vs. 144.0±14.01% NHE1; *P*< 0.05). Our findings revealed for the first time that NHE1 induced OPN expression regulated hypertrophic gene transcription.

### OPN regulates NHE1 activity

**Fig 4:**
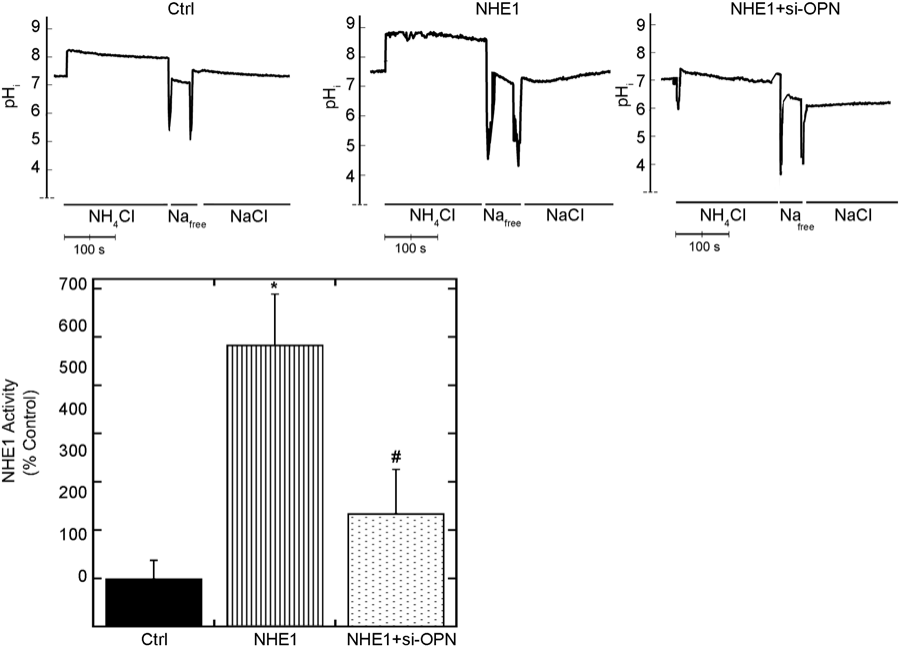
Downregulating OPN reduces NHE1-induced cardiomyocyte-hypertrophy and NHE1 activity. Cardiomyocytes plated on coverslips were incubated with BCECF-AM and induced with an acid load using 50 mM NH_4_Cl. The rate of recovery following the acid induction was measured and used as an indicator of NHE1 activity. Upper panel, representative traces of NHE1 activity assay in H9c2 cardiomyocytes infected with GFP (control) or NHE1 in the presence and absence of siRNA OPN for 24 hours; lower panel, quantification of NHE1 activity (10–14 coverslips, from 3–4 experiments). Results are expressed as % of control (GFP) ± %SEM. *p < 0.05 vs. control, # vs. NHE1.

To ascertain the involvement of OPN on enhancing/maintaining NHE1 activity, NHE1 activity was measured in cardiomyocytes expressing active NHE1 in the presence and absence of OPN siRNA (Fig 4). NHE1 activity in cardiomyocytes infected with active NHE1 was significantly increased compared to control (586.5±103.54% of control; *P*<0.05). NHE1 activity was reduced by more than 75% by downregulating OPN expression in cardiomyocytes (235.0±92.14% vs. 586.5±103.54% NHE1; *P*< 0.05). These data demonstrated that the expression of OPN in cardiomyocytes contributed to NHE1 activity suggesting that OPN played a role in enhancing/maintaining NHE1 activity.

### RSK facilitates the NHE1 induced OPN expression

**Fig 5:**
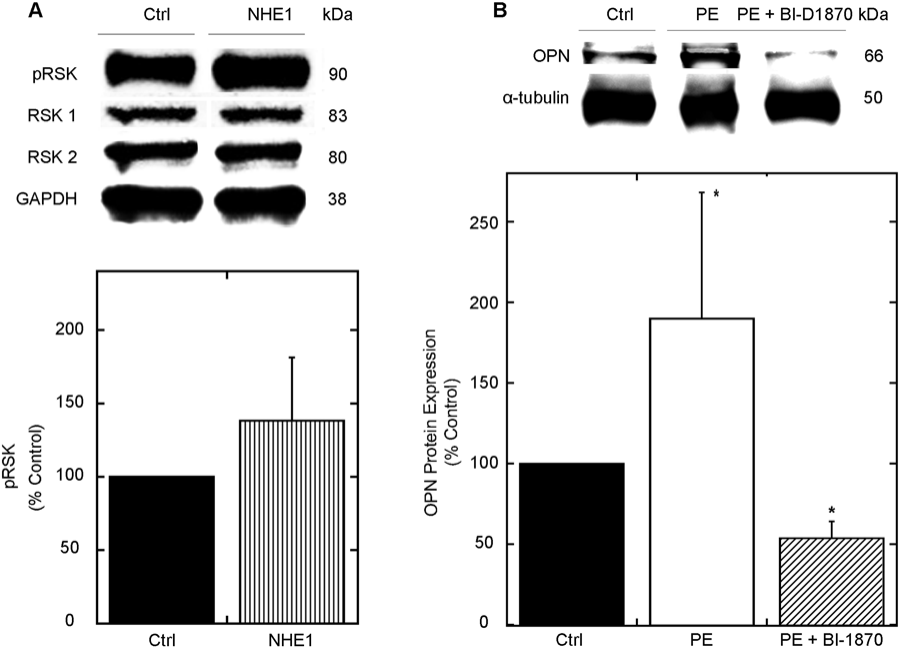
OPN contributes to NHE1-induced cardiomyocyte-hypertrophy through RSK. A. Upper panel: Representative western blot of relative amounts of phosphorylated and total expression of RSK in NRVMs infected with GFP (control) or active NHE1. Immunoblotting was against *phosphorylated* and *total* RSK (80–90 kDa) and normalized to GAPDH (38 kDa); lower panel, quantification of experiments measuring the ratio of phosphorylated to total protein for RSK. Results are expressed as % of control (GFP) ± %SEM (n = 4; representative of 2 preparations). B. H9c2 cardiomyoblasts were treated with PE and/or BI-D1870 (10 μM) for 24 h. Cells were lysed and equal amounts of protein were analyzed by SDS-PAGE/immunoblot Immunoblotting was against OPN (doublet at 66 kDa) and α-tubulin (50 kDa); lower panel, quantification of relative levels of OPN protein expression (n = 4–5; representative of 5 experiments). Results are expressed as % of control ± %SEM. *p < 0.*05* vs. control.

Several kinases including ERK 1/2, RSK and Akt have been shown to be induced during conditions of CH [42]. Whether NHE1 induced OPN expression in cardiomyocytes is mediated in part by the MAPK signaling pathway has not been demonstrated. To further understand how active NHE1 increases OPN expression in cardiomyocytes, we measured the expression of the phosphorylated and total proteins of ERK 1/2 (S4 Fig), Akt (S4 Fig) and RSK (Fig 5A) following 24 hours of infection with the active NHE1 adenovirus. The ratio of phosphorylated to total ERK 1/2 and Akt were not significantly different in cardiomyocytes expressing active NHE1 (S4 Fig). Similarly, cardiomyocytes infected with the active form of the NHE1 adenovirus demonstrated a trend towards increase in the ratio of phosphorylated to total RSK (138.2±43.02% of control), but not a significant increase. Further studies were carried out to delineate the effects of RSK in the NHE1-induced OPN hypertrophic response following a time dependent stimulation with PE, a known NHE1 stimulator [43], in the presence of BI-D1870 (Fig 5B). Our findings revealed that PE induced the expression of OPN following 30 minutes of PE stimulation, an effect that was significantly reduced in the presence of BI-D1870 (53.7±10.45% vs. of control; *P*< 0.05). These results suggested that the NHE1 induced OPN expression may in part be dependent on RSK.

## Discussion

The expression of constitutively active NHE1 and OPN have both been shown to individually contribute to the development and progression of CH [5,15]. Recently, the upregulation of NHE1 in CH models was correlated with an increase in OPN expression. However, no reports have directly investigated whether the expression of active NHE1 induces OPN and how OPN contributes to the NHE1 mediated hypertrophic response in cardiomyocytes. This study demonstrates demonstrated that OPN mRNA and protein expression are significantly increased in cardiomyocytes expressing active NHE1, which in part maybe attributed to RSK.

In our study, H9c2 the myoblast cell line, derived from embryonic BD1X rat heart tissue [44] in their differentiated form as cardiomyocytes [34] was used to block OPN expression induced by infection with the active form of the NHE1 adenovirus. H9c2 cells have been shown to display hypertrophy-associated traits comparable to primary cultures of cardiomyocytes when stimulated with α1-adrenergic receptor agonists as well as high glucose treatment [32,33]. As such, we chose to investigate the effects of downregulating OPN on NHE1-induced cardiomyocyte hypertrophy in H9c2 cardiomyoblasts. In order to develop a more representative model, H9c2 cardiomyoblasts were differentiated into the cardiac phenotype using retinoic acid. [34]. Retinoic acid has been shown to stimulate the expression of cardiac specific L-type voltage-dependent Ca^2+^ channels (VDCCs), the cardiac sarcomeric heavy chain and myosin light chains, which are parameters specific to the cardiac muscles cells [34,45,46]. Our choice of H9c2 cardiomyocytes seemed more suited for our experiments with siRNA, since they represent a form of immortal mammalian cell line, which are easier to transfect [41] as oppose to NRVMs [31]. Moreover, we anticipated that siRNA OPN would be more effective at downregulating the expression of OPN in H9c2 cardiomyocytes since they originate from myoblasts [47,48].

OPN is differentially expressed as two isoforms, a *secreted* full length OPN (sOPN) and an *intracellular* (iOPN) form [49]. sOPN, a matricellular protein of the extracellular matrix, binds to the cell surface proteoglycans [50]. A large portion of the sOPN remains associated with the surface of the extracellular matrix making it rather difficult to detect changes in the media [51,52]. As a result, in the majority of *in vitro* studies [39,51–53] and in our study, OPN expression was examined in the cell lysates, where we demonstrated an increase in OPN mRNA and protein expression, in agreement with previous findings [54] [55,56]. The increase in OPN expression appeared to occur in both NRVMs and H9c2 cardiomyocytes.

Cardiomyocytes expressing the active form of the NHE1 adenovirus significantly increased cell surface area, protein content and ANP mRNA (Fig 2A–2D), confirming previous reports, which have demonstrated that active NHE1 induces cardiomyocyte hypertrophy [11,26,30]. Cell area was measured in both H9c2 cardiomyocytes and NRVMs following infection with the NHE1 adenovirus and in both models, a significant increase in cell area was observed. For the first time our group demonstrated that the NHE1-mediated increase of cell surface area, protein content and ANP mRNA were significantly reduced upon down regulation of OPN. Furthermore, our findings indicated that the upregulation of OPN alone in cardiomyocytes infected with the OPN adenovirus was not sufficient to induce cardiomyocyte hypertrophy. Taken together, our results suggest that OPN is permissive for the hypertrophic effects of active NHE1 in cardiomyocytes, although it is not sufficient to induce cardiomyocyte hypertrophy without NHE1.

Mraiche et al. demonstrated that transgenic mice expressing wild type NHE1 induced lower expression of OPN and the hypertrophic response compared to transgenic mice expressing activated NHE1 [5]. This highlighted the importance of active NHE1 in OPN induction. Interestingly, overexpression of OPN alone in cardiomyocytes was not sufficient to significantly induce a cardiomyocyte hypertrophy phenotype, further supporting the importance of the expression of active NHE1 in inducing the upregulation of OPN in cardiomyocyte hypertrophy.

The importance of NHE1 in inducing OPN has also been observed in conditions of metastasis; where the activity of the α_v_β_3_ integrin, an osteopontin receptor whose activity is regulated by NHE1, was elevated thus allowing cell activation by OPN [57,58]. mRNA expression of CD44, a receptor that also interacts with and causes the activation of OPN, was also shown to be significantly elevated in transgenic mice expressing active NHE1 and exhibiting an upregulation of OPN [59].

The expression of OPN has previously been shown to be upregulated in response to the activation of the CaN/NFAT pathway [60], which coincides with the enhanced nuclear translocation of the GATA4 transcription factor [17,23]. In our study, enhanced expression and activity of NHE1 caused the exacerbation of cardiomyocyte hypertrophy, which is associated with the activation of GATA4. This is in agreement with previous studies, which have shown that the activation of NHE1 and cardiomyocyte hypertrophy were associated with activation of the CaN/NFAT and GATA4 signaling [25,61]. In our study, we demonstrated for the first time that NHE1 induced OPN mediated the phosphorylation of GATA-4, which is reflected by the decreased expression of the phosphorylated form of GATA4 in H9c2 cardiomyocytes co-infected with the active form of the NHE1 adenovirus and siRNA-OPN. However, the direct role of the CaN/NFAT pathway in the NHE1 induced OPN hypertrophic response remains unknown.

In the myocardium, RSK is considered a primary regulator of NHE1 activity through phosphorylation of the Ser^703^ site along the C-terminal, thereby facilitating the binding of the 14-3-3 protein to the phosphorylated Ser^703^ residue [62]. In cardiac tissue from SGK1^+/+^ mice, the phosphorylation of NHE1, as indicated by NHE1 immunoprecipitated samples using an anti-phospho- (Ser) 14-3-3 antibody, was significantly higher compared to KO mice following dexamethasone stimulation [63]. Several other kinases including ERK 1/2, Akt have been shown to regulate NHE1 activity [8,28,29] as well as OPN expression in the setting of cardiomyocyte hypertrophy [27,64]. Our findings suggest, as indicated by the ratio of phosphorylated to total forms of the respective kinase protein expression, that following 24 hours of infection of cardiomyocytes with NHE1, no significant changes in ERK ½, Akt or RSK expression was observed (S4 Fig and Fig 5A). This may in part be due to the timing of our experiments, which were carried out 24 hours post-transfection with the NHE1 adenovirus. Our findings may also be attributed to the lower number of NRVM preparations used in this set of experiments. Nakamura et al. demonstrated that the hypertrophic effects induced by enhanced activity of NHE1 occurred independent of Akt activation, which is in agreement with our findings [30]. In addition, a recent study has shown that CU-NP, a non-vasodilating natriuretic peptide, was able to inhibit and reverse NHE1-induced cardiomyocyte hypertrophy independent of ERK 1/2 pathway [65]. Our findings also appear to be consistent with previous reports that have suggested a link between NHE1 and RSK in mediating the hypertrophic response [28,29]. Interestingly, previous reports have also identified an association between RSK and OPN. Inhibition of RSK *in vitro* has been previously associated with a downregulation in OPN expression [21]. In fact, RSK-induced activation of OPN-CD44 receptor in epithelial cells has been shown to be modulated upon activation of the MEK and RSK pathways, all of which were decreased in the presence of FMK, a RSK inhibitor [66]. In our study, the inhibition of RSK through BI-D1870 reduced the NHE1-induced OPN expression following PE (30 minutes) stimulation (Fig 5B) suggesting that the NHE1-induced OPN upregulation may in part be mediated through RSK. However, further studies are required to elucidate the role of RSK in the NHE1 induced OPN hypertrophic cascade.

In this study, we demonstrate that OPN is able to facilitate the NHE1-induced hypertrophic response using an *in vitro* model. Several studies have documented the importance of OPN in the induction of both CH [17] and dilated cardiomyopathy [18] *in vivo*. Further studies will be necessary to examine the role of NHE1-induced OPN in a model in which cardiomyocytes and fibroblasts are co-cultured. It will also be necessary to determine the cellular interplay between OPN and NHE1 in an *in vivo* model of cardiac hypertrophy. Overall our data demonstrates that the enhanced expression and activity of NHE1 causes an upregulation of OPN, which in turn contributes to the hypertrophic response elicited in cardiomyocytes. Interestingly, downregulation of OPN successfully reverted the hypertrophic response and decreased NHE1 activity.

## Supporting Information

S1 Fig Multiplicity of infection (MOI) of NHE1 adenoviral infection in NRVMs. Immunoblotting was against anti-HA tag for exogenous NHE1 (90–110 kDa), total NHE1 (90–110 kDa) or GAPDH (38 kDa). A: Representative western blot of NRVMs infected with control (GFP) or active NHE1 adenovirus at an MOI of 10, 20 or 30, respectively for 24 hours and probed against the anti-HA tag antibody (n = 3). B: Representative western blot of NRVMs infected with control (GFP) or active NHE1 adenovirus at an MOI of 10, 20 or 30, respectively for 24 hours and probed against the anti-NHE1 antibody (n = 3).
(TIF)

S2 Fig MOI of NHE1 adenoviral infection in H9c2 cardiomyocytes. Immunoblotting was against anti-HA tag for exogenous NHE1 (90–110 kDa), total NHE1 (90–110 kDa) or GAPDH (38 kDa) protein expression. A: Representative western blot of H9c2 cardiomyocytes infected with control (GFP adenovirus) using an MOI of 20 or 30 or NHE1 adenovirus using an MOI of 10, 20 or 30, respectively for 24 hours and probed against the anti-HA tag antibody (n = 3). B: Representative western blot of H9c2 cardiomyocytes infected with control (GFP) using an MOI of 20 or 30 or active NHE1 adenovirus using an MOI of 10, 20 or 30 for 24 hours and probed against the anti-NHE1 antibody (n = 3).
(TIF)

S3 Fig OPN protein expression is greatest at 24 hours compared to 6 and 12 hours in H9c2 cardiomyocytes. A: OPN protein expression in H9c2 cardiomyocytes at 2, 6, 12 and 24 hours after collecting cell lysates. Immunoblotting was against anti- OPN for *total* OPN (doublet at 66 kDa) and α- tubulin (50 kDa) (n = 3). B: Representative western blot of total OPN protein expression of H9c2 cardiomyocytes infected with GFP 20 MOI, OPN 30 MOI, 30 nM universal scrambled siRNA or 100 nM siRNA OPN for 24 hours. Immunoblotting was against anti- OPN for *total* OPN (doublet at 66 kDa) and α- tubulin (50 kDa) (n = 3).
(TIF)

S4 Fig ERK 1/2 and Akt are not implicated in NHE1-induced OPN expression and cardiomyocyte-hypertrophy. Representative western blot of relative amounts of phosphorylated and total protein expression of Akt and ERK 1/2 in NRVMs infected with GFP (control) or active NHE1. Immunoblotting was against *phosphorylated* and *total* Akt (60 kDa) or ERK 1/2 (43–44 kDa) and normalized to GAPDH (38 kDa).
(TIF)
