# Supplementary figures and images for: Na+/H+ Exchanger Isoform 1-Induced Osteopontin Expression Facilitates Cardiomyocyte Hypertrophy
Source: PLoS One. 2015 Apr 17;10(4):e0123318. doi: 10.1371/journal.pone.0123318 (PMC4401699; doi:10.1371/journal.pone.0123318)

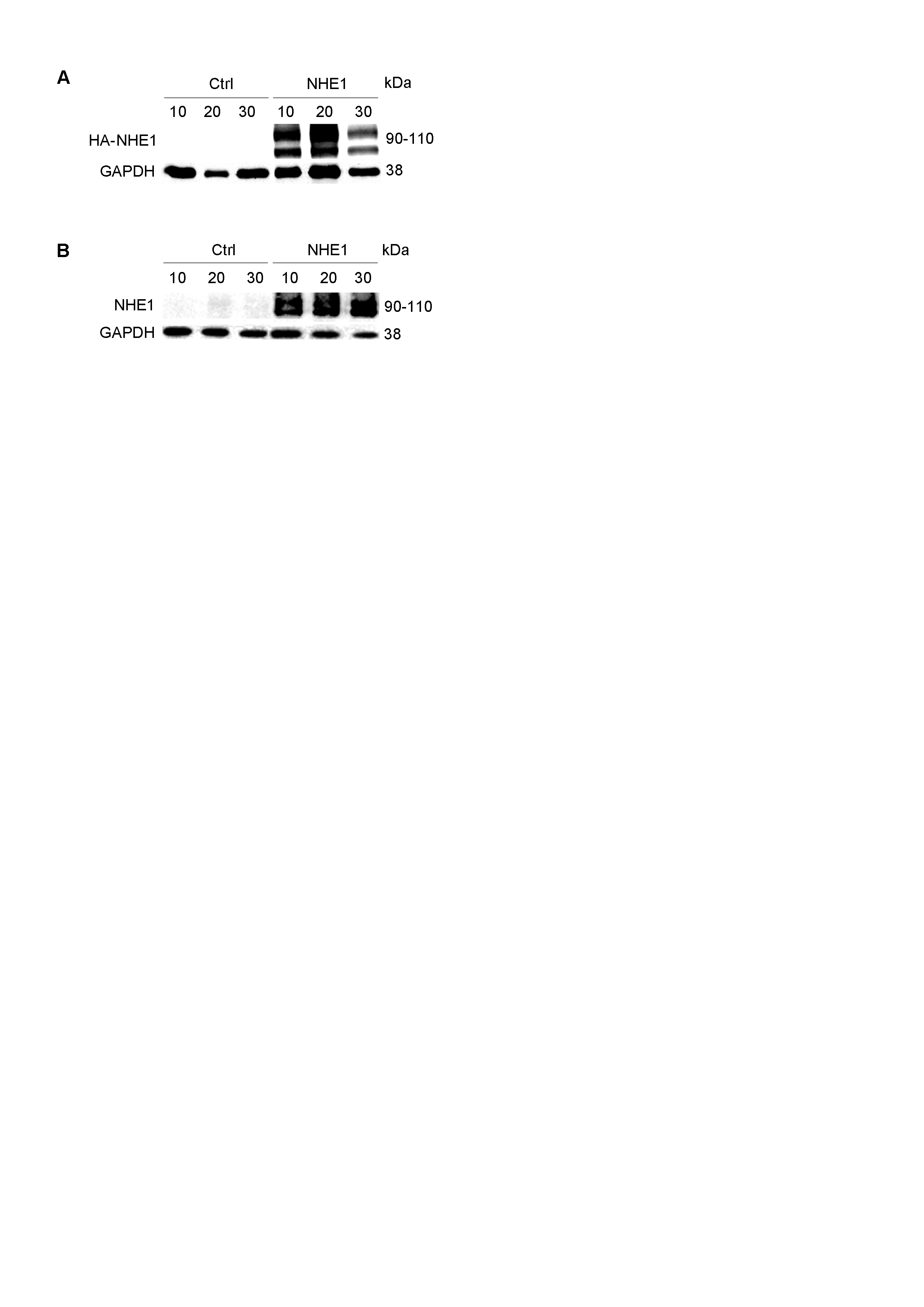

Supplement: S1 Fig — Immunoblotting was against anti-HA tag for exogenous NHE1 (90–110 kDa), total NHE1 (90–110 kDa) or GAPDH (38 kDa). A: Representative western blot of NRVMs infected with control (GFP) or active NHE1 adenovirus at an MOI of 10, 20 or 30, respectively for 24 hours and probed against the anti-HA tag antibody (n = 3). B: Representative western blot of NRVMs infected with control (GFP) or active NHE1 adenovirus at an MOI of 10, 20 or 30, respectively for 24 hours and probed against the anti-NHE1 antibody (n = 3). (TIF) [file pone.0123318.s001.tif]

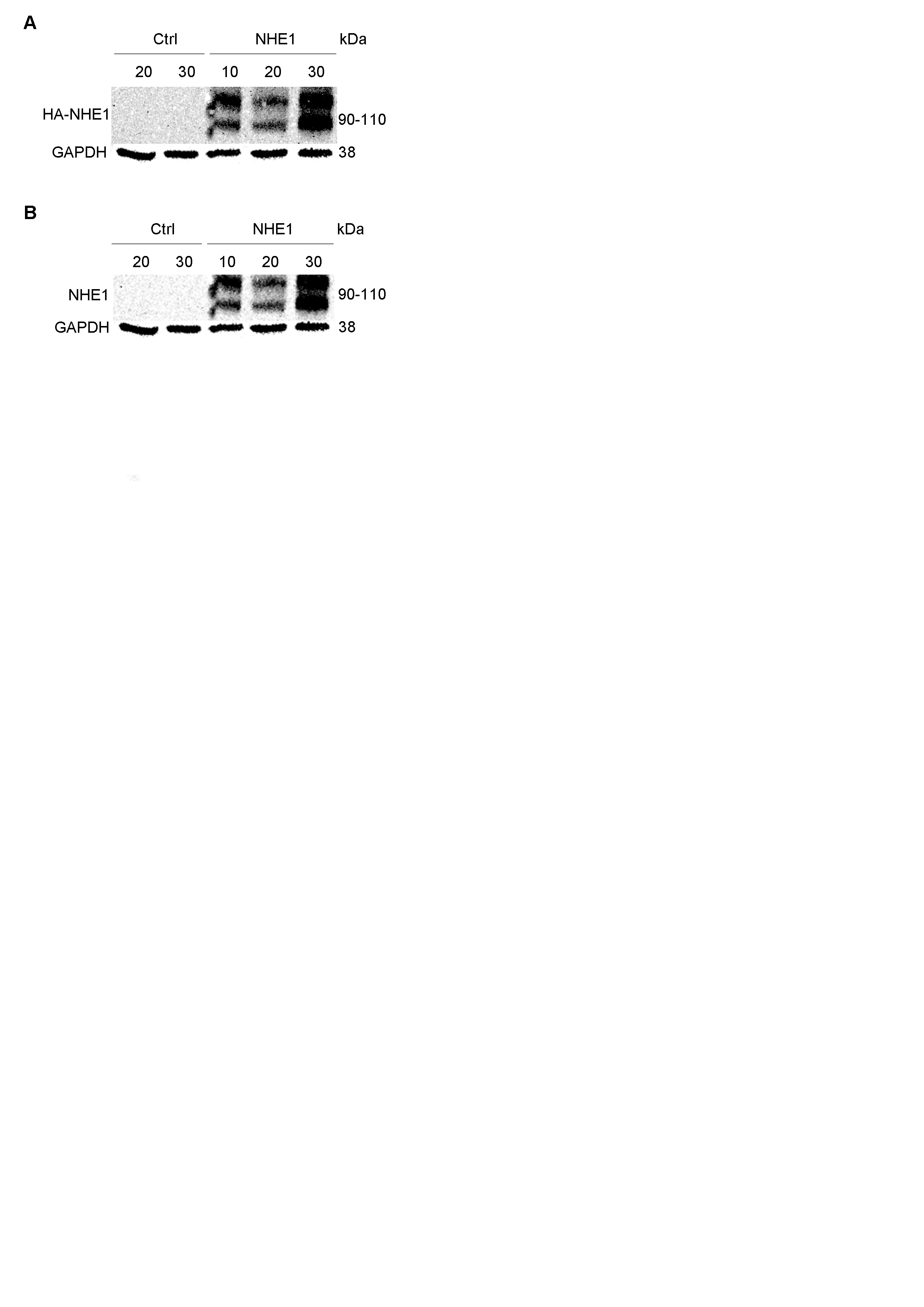

Supplement: S2 Fig — Immunoblotting was against anti-HA tag for exogenous NHE1 (90–110 kDa), total NHE1 (90–110 kDa) or GAPDH (38 kDa) protein expression. A: Representative western blot of H9c2 cardiomyocytes infected with control (GFP adenovirus) using an MOI of 20 or 30 or NHE1 adenovirus using an MOI of 10, 20 or 30, respectively for 24 hours and probed against the anti-HA tag antibody (n = 3). B: Representative western blot of H9c2 cardiomyocytes infected with control (GFP) using an MOI of 20 or 30 or active NHE1 adenovirus using an MOI of 10, 20 or 30 for 24 hours and probed against the anti-NHE1 antibody (n = 3). (TIF) [file pone.0123318.s002.tif]

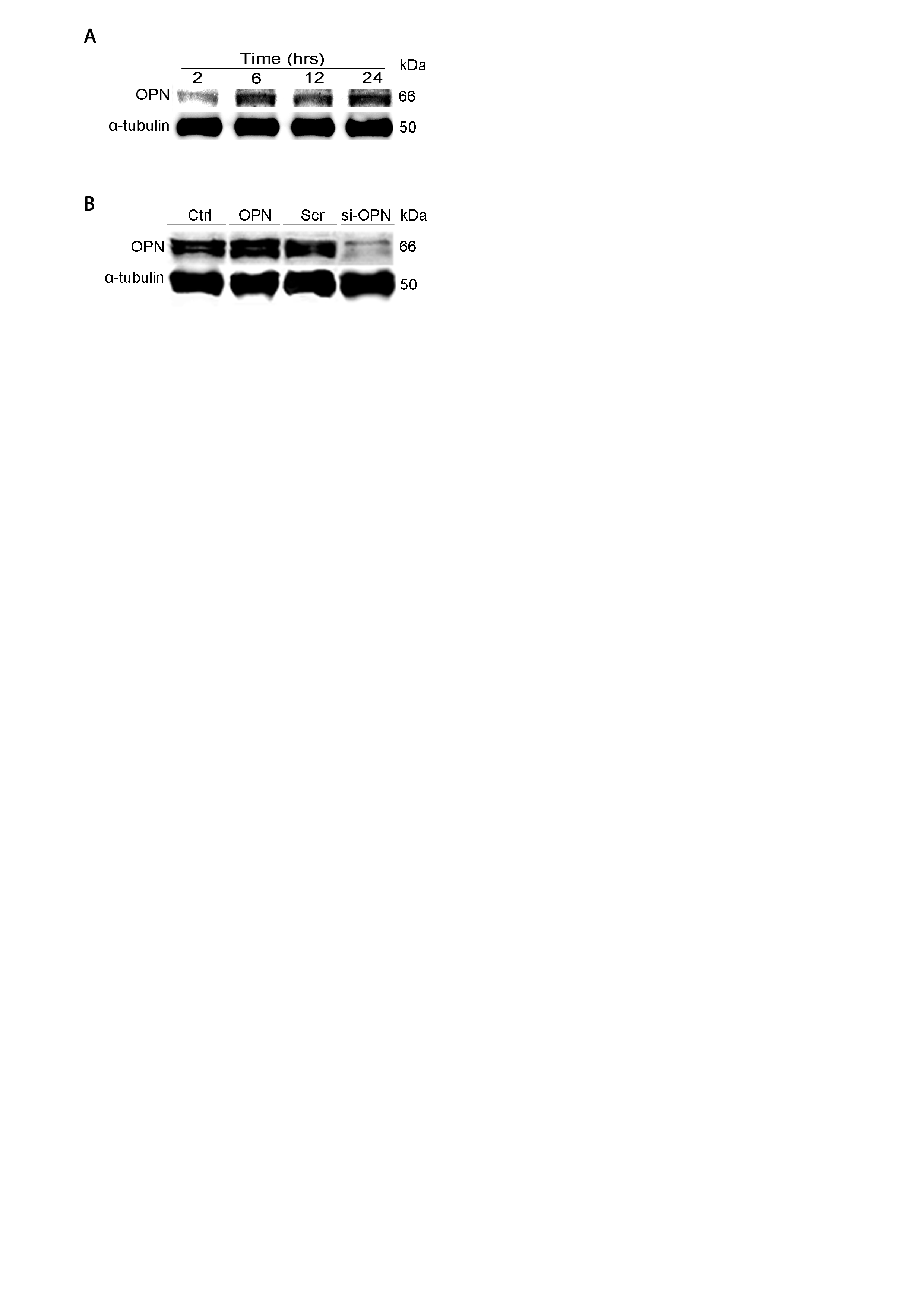

Supplement: S3 Fig — A: OPN protein expression in H9c2 cardiomyocytes at 2, 6, 12 and 24 hours after collecting cell lysates. Immunoblotting was against anti- OPN for total OPN (doublet at 66 kDa) and α- tubulin (50 kDa) (n = 3). B: Representative western blot of total OPN protein expression of H9c2 cardiomyocytes infected with GFP 20 MOI, OPN 30 MOI, 30 nM universal scrambled siRNA or 100 nM siRNA OPN for 24 hours. Immunoblotting was against anti- OPN for total OPN (doublet at 66 kDa) and α- tubulin (50 kDa) (n = 3). (TIF) [file pone.0123318.s003.tif]

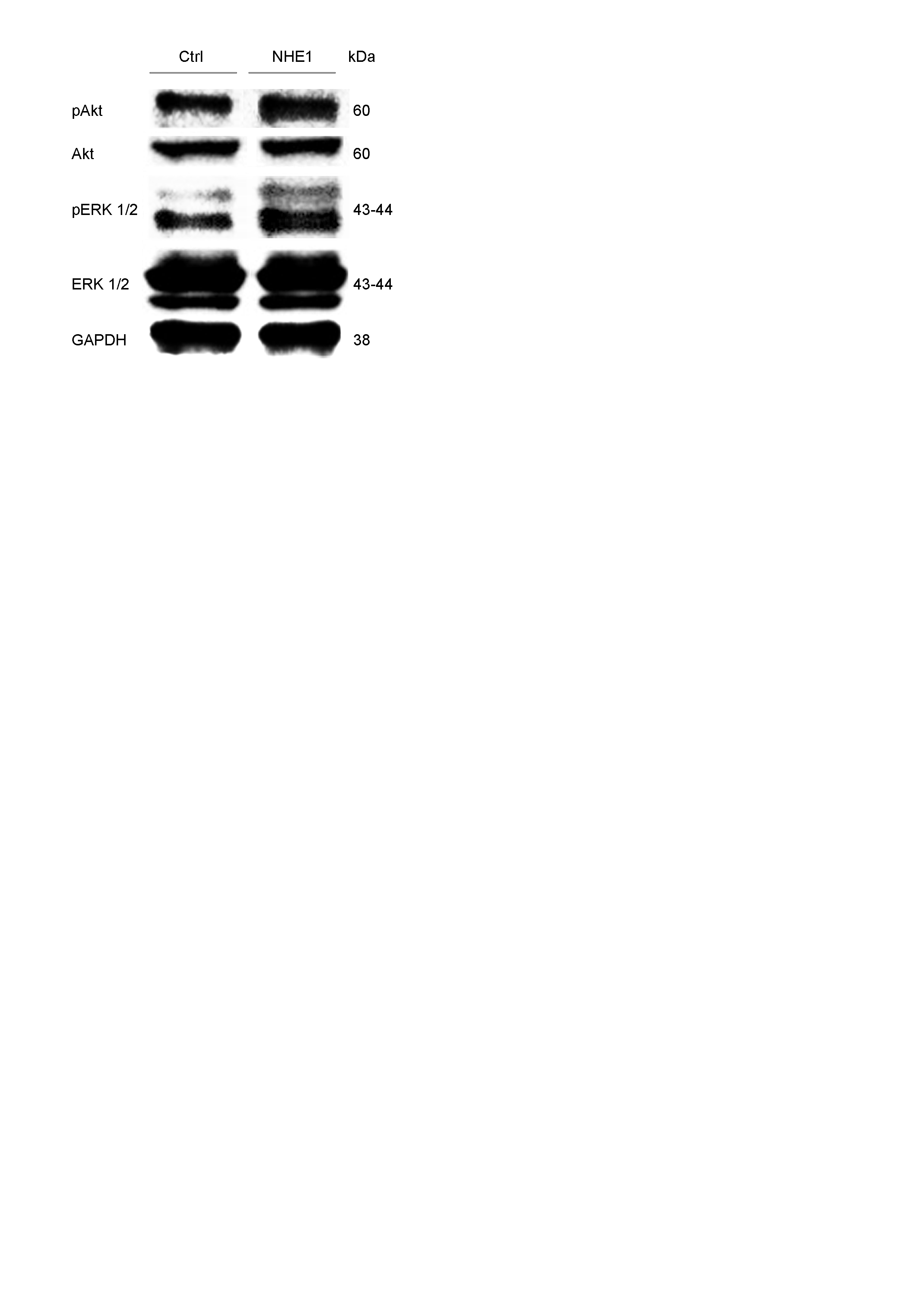

Supplement: S4 Fig — Representative western blot of relative amounts of phosphorylated and total protein expression of Akt and ERK 1/2 in NRVMs infected with GFP (control) or active NHE1. Immunoblotting was against phosphorylated and total Akt (60 kDa) or ERK 1/2 (43–44 kDa) and normalized to GAPDH (38 kDa). (TIF) [file pone.0123318.s004.tif]
